# The Response of Metallothionein and Malondialdehyde after Exclusive and Combined Cd/Zn Exposure in the Crab *Sinopotamon henanense*

**DOI:** 10.1371/journal.pone.0080475

**Published:** 2013-11-18

**Authors:** Yingjun Li, Xi Chai, Hao Wu, Weixin Jing, Lan Wang

**Affiliations:** School of Life Science, Shanxi University, Taiyuan, China; Queen Mary University of London, United Kingdom

## Abstract

The purpose of this paper is to show the interactions of Cd and Zn in the freshwater crab *Sinopotamon henanense* through metallothionein (MT) and malondialdehyde (MDA) level measurements. Laboratory acclimated *S.henanense* were exposed to Cd (50 µg/L, 100 µg/L, 500 µg/L ), and Zn (100 µg/L, 1000 µg/L) alone and in combined treatments (100 µg/L Zn+50 µg/L Cd, 100 µg/L Zn+100 µg/L Cd, 100 µg/L Zn+500 µg/L Cd, 1000 µg/L Zn+50 µg/L Cd, 1000 µg/L Zn+100 µg/L Cd, 1000 µg/L Zn+500 µg/L Cd) for 7, 14, 21, 28, 35 days. The results demonstrated that the MDA contents increased with exposure time and dose and showed time- and dose-dependence in both gills and hepatopancreas of *S.henanense* after single Cd exposure, while the changes of MDA levels were not significant with single Zn exposure. The MDA levels decreased when the crabs were exposed to metal mixtures compared to Cd exposure alone, indicating that Zn mediated the cellular toxicity of Cd. MT contents increased after single Cd exposure and also showed a time- and dose-dependence, in a tissue-specific way. Zn showed a limited ability of MT induction both in gills and hepatopancreas of *S.henanense*. The MT contents represented not a simple addition of single metal exposures but were enhanced at a higher concentration of Zn combined with different Cd concentrations compared to single metal exposure. Whether MT can be used as a biomarker for complex field conditions need to be considered cautiously since different induction patterns of MT were found among single Zn, Cd and combined groups. It is suggested that several biomarkers together as a suite should be used in the monitoring of heavy metal pollution in the aquatic environment.

## Introduction

Man-made pollution provides simultaneous environmental contamination with a multitude of xenobiotics. The investigation of combined toxicity effects of multiple pollutants is, therefore, demanded in the monitoring and prediction of chemical impacts. However, most toxicological studies focus on the effects of a single xenobiotic as yet [1], [2], [3].

In general, the combination of two or more pollutants may result in any of the following effects: the addition of single chemical toxic effects (additive effect); toxic effects of the mixture being significantly less than the sum of single chemical toxic effects (antagonism); toxic effects of the mixture significantly exceeding the sum of single chemical toxic effects (synergism). Since environmental conditions result from the interaction of complex array of xenobiotics the understanding of the underlying mechanism of combined xenobiotic toxicity is crucial for environmental risk assessments and their interactions need to be studied. This also holds for heavy metals. Among them are cadmium (Cd) and zinc (Zn) contaminations ubiquitous in the environment and found increasing attention by ecotoxicologists in recent years. Interestingly, the results of such attempts are in consistant in most cases. For example, interacts Cd synergistically with Zn in the amphipod *Corpophium volutator* [4], [5]. However, antagonistic interactions have been found with the same trace metal combination for the prawn *Palaemon serratus* [6].

To investigate further this controversial issue, we used the freshwater crab *Sinopotamon henanense*as a model. This crab is widely distributed in northern China and can easily be obtained and cultured in the laboratory. It is also an important link in the food web [7], [8], [9]. *S.henanense* is thereby a useful test organism to investigate the impacts of pollutants, including metals. Several studies have been focusing on the toxic effects of Zn and Cd in crabs through single metal exposure in the past years [10], [11], [12], [13], [14]. However, only little is known about combined effects and interactions of these metals in crabs [5], [15].

Metallothioneins (MTs), with low molecular weight, cysteine-rich protein, are the most abundant intracellular metal-binding proteins. MT is induced by heavy metals and binds metal ions for metal detoxification or regulation in organisms [16]. Due to a dose response was exhibited, the MT concentration in the crabs have been considered as a heavy metal exposure biomarker in several recent studies [8], [12], [17], [18], [19], [20], [21].

The present study will focus on the response of *S.henanense* exposed for 35 days to waterborn contamination with the essential trace metal Zn and the non-essential metal Cd in single and combined applications. The objective of the current work was to investigate the toxic effects of single and co-exposure of Cd and Zn in order to understand the mechanisms of Cd-Zn interactions.

## Materials and Methods

### Chemicals

All chemicals used in the present study were analytical grade, obtained from Sigma Co. (St. Louis, MO).

### Experimental animals and treatments

Freshwater crabs (*S*.*henanense*) were purchased from Taiyuan Dong-an aquatic product wholesale market in Shanxi province, China. Only healthy adult crabs of similar weight were used for these experiments. Before experimentation the crabs were acclimated in the laboratory for more than two weeks in a glass aquarium filled with dechlorinated water, at a light regime of 12h light/12h dark and a temperature of 20±2°C. The aquarium was shielded by a black plastic to reduce disturbance. Crabs were fed every two days with commercial fish pellet feeds, uneaten feeds were removed at the end of the day, and water was exchanged every three days and the glass aquarium was cleaned thoroughly.

After acclimation, crabs were randomly numbered and divided into twelve experimental groups of four animals each, providing a control group and metal exposure groups with either Zn^2+^or Cd^2+^ separately, and Zn^2+^ combined with Cd^2+^. The exposure protocol was: control, 50 µg/L CdCl_2_, 100 µg/L CdCl_2_, 500 µg/L CdCl_2_, 100 µg/L ZnSO_4_, 1000 µg/L CdCl_2_, 50 µg/L CdCl_2_+100 µg/L ZnSO_4_, 100 µg/L CdCl_2_+100 µg/L ZnSO_4_, 500 µg/L CdCl_2_+100 µg/L ZnSO_4_, 50 µg/L CdCl_2_+1000 µg/L ZnSO_4_, 100 µg/L CdCl_2_+1000 µg/L ZnSO_4_, 500 µg/L CdCl_2_+1000 µg/L ZnSO_4_, the crabs were exposed for 7, 14, 21, 28, 35days in glass aquaria, and were fed every two days during the experimental period. The water was changed thoroughly every two days during the exposure regime, all other conditions were kept the same as those used for acclimation.

### MDA content determination in the gills and hepatopancreas of *S.henanense*

Levels of LPO were measured by the generation of thiobarbituric acid (TBARS) which are referred to as malondialdehyde (MDA) reactive species equivalents. Color development due to the reaction of thiobarbituric acid reactive substances (TBARS) with MDA was measured at 532 nm using a SpectraMax M5 to determine levels of LPO by following the method of Ohkawa et al. [22] and expressed as nanomoles of malondialdehyde released/mg protein.

### MT level measurement in the gills and hepatopancreas of *S.henanense*

The concentration of MT was determined by a Cadmium-saturation assay based on the method of Pedersen et al. [23] and He et al. [24]. The procedure used was similar to that described by Ma et al. [8] with slight modification. Approximately 1g wet weight gill sample was homogenized in 4ml 10mM Tris-HCl buffer (pH8.0) with an electric homogenizer. The homogenization buffer also contained 0.1 mM phenylmethyl sulphonyl fluoride (PMSF) and 0.1 mM dithiothreitol (DTT). The homogenate fluid was centrifuged at 12000×g for 30 min at 4°C, and the supernatant was heated for 2 min at 100°C using a water-bath. The heated samples were centrifuged at 12000×g for 10 min to remove precipitated proteins. Each 0.5 mL sample (heat-denatured supernatant) was mixed with 0.1 mL Cd solution (500 µg L-1 as CdCl2) and incubated at room temperature for 10 min to saturate the metal binding sites of MT. A 2% (w/v) bovine hemoglobin solution (Sigma Com.) was used to add 0.5 mL and incubate the mixture at room temperature for 10 min. The hemoglobin was denatured in a water bath (100°C) for 3 min, cooled on ice for 5 min, and centrifuged at 12000×g for 15 min. The denatured proteins, except for MT which is heat stable, were removed by centrifugation. Steps from the addition of the bovine hemoglobin solution until centrifugation were repeated two more times. An atomic absorption spectrophotometer (Varian AA240FS, USA) was used to analyze the concentration of cadmium. The estimated concentration of MT was calculated by the following equation:
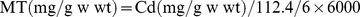



In theory, one MT molecule could combine with six Cd^2+^, and the concentration of MT in gills was calibrated accordingly [25], [26].

### Protein concentration

The protein concentration was determined using the Bradford [27] assay method, with bovine serum albumin as a standard.

### Statistical analysis

Statistical analysis was performed using SPSS 15.0 software. All the values are expressed as mean±standard deviation (SD). The data from animals exposed to Cd^2+^, Zn^2+^, or Zn^2+^ plus Cd^2+^ were compared with those from control animals. Statistical analysis was carried out using one-way analysis of variance (ANOVA) to evaluated whether the means were significantly different, and significance was tested at* *P*<0.05, and** *P*<0.01.

## Results

### MDA contents in the gills and hepatopancreas of *S. henanense*

**Figure 1:**
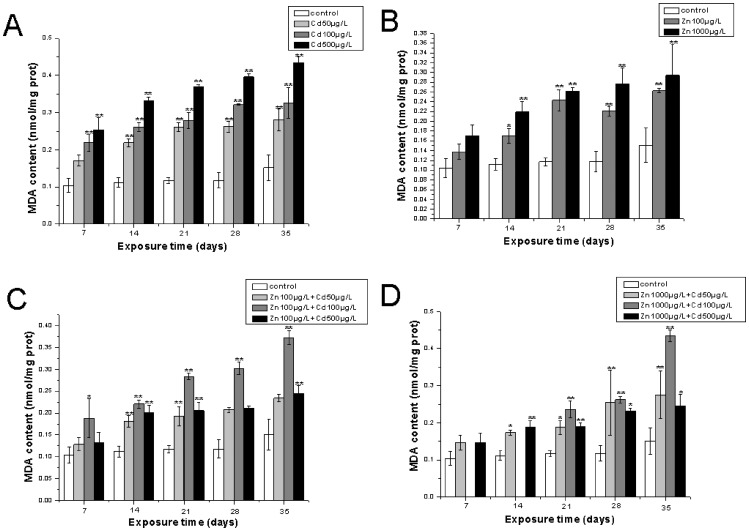
The MDA contents in the gills of *S.henanense* exposed to Cd and Zn either as a separate treatment or both metals combined for 7, 14, 21, 28, 35days. Data were expressed as mean values ±standard error. Statistical significances were analyzed using one-way ANOVA compared to the control, *P<0.05, **P<0.01.

**Figure 2:**
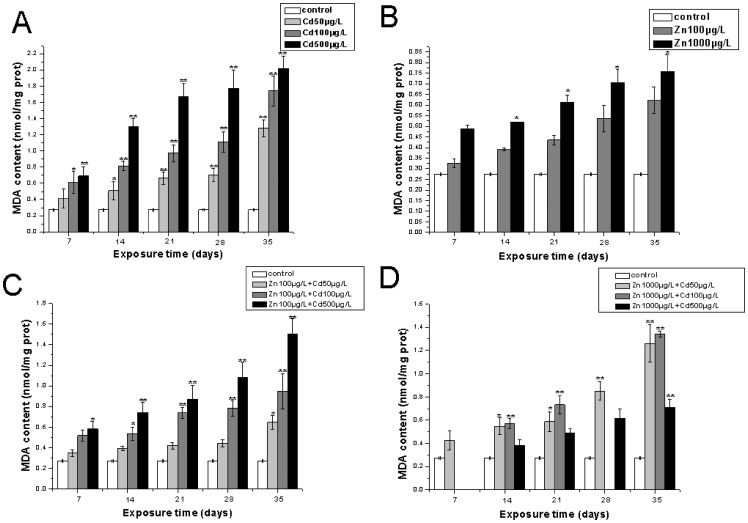
The MDA contents in the hepatopancreas of *S.henanense* exposed to Cd and Zn either as a single treatment or both metals combined for 7, 14, 21, 28, 35days. Data are expressed as mean values ±standard error. Statistical significances were analyzed using one-way ANOVA compared to the control, *P<0.05, **P<0.01.

The levels of MDA in gills and hepatopancreas of *S.henanense* were shown in Fig.1 and Fig.2, respectively. In the treatment of crabs exposed to single Cd, there was a steady time and dose-dependent increase in the levels of MDA, both within gills and hepatopancreas throughout the experimental period, and the MDA level reached highest values, about 0.43 nmol/mg protein after exposure for 35 days when treated with 500 µg/LCdCl_2_ within gills (Fig.1A), the highest value of 2.02±0.15 nmol/mg protein was observed in the hepatopancreas of *S.henanense* with the same treatment as in gills (Fig.2A). In the treatment with single Zn, the amount of MDA increased slowly until day 35, and the highest value of 0.29±0.062 nmol/mg protein was reached with the exposure of 1000 µg/L Zn for 35 days in the gills of *S.henanense* (Fig.1B). The MDA levels accelerated more gently within the hepatopancreas than in gills and reached the highest value of 0.76±0.078 nmol/g protein with the treatment of 1000 µg/L Zn for 35 days (Fig.2B). The MDA elevated concentrations with single Zn treatments were lower than with single Cd exposures both in the gills and hepatopancreas as seen in Fig.1 and Fig.2. The resulting MDA concentrations of treatments with 100 µg/L Zn, 1000 µg/L Zn adding different doses of Cd in gills were shown in Fig. 1C and Fig. 1D, respectively. The MDA concentration increased gradually till the end of the experimental regime. Interestingly, the highest value appeared in the middle metal mixture groups and reached 0.37±0.1 nmol/ mg protein and 0.43±0.017 nmol/ mg protein in the treatment with 100 µg/L Zn +100 µg/L Cd and 1000 µg/L Zn +100 µg/L Cd for 35 days, respectively, in the gills (Fig.1C; Fig.1D). The change of MDA levels was tissue-specific. The MDA concentration increased with time and dose in the treatment of Cd/Zn co-exposure groups where 100 µg/L Zn were added in the hepatopancreas (Fig.2C), whereas in the hepatopancreas of *S.henanense*a similar phenomenon can be seen in the treatment of metal mixtures, where 1000 µg/L Zn was added in the gills (Fig.2D). The data obtained showed that the MDA content in the metal mixture group was lower than a single Cd treatment.

### MT levels in the gills and hepatopancreas in *S. henanense*

**Figure 3:**
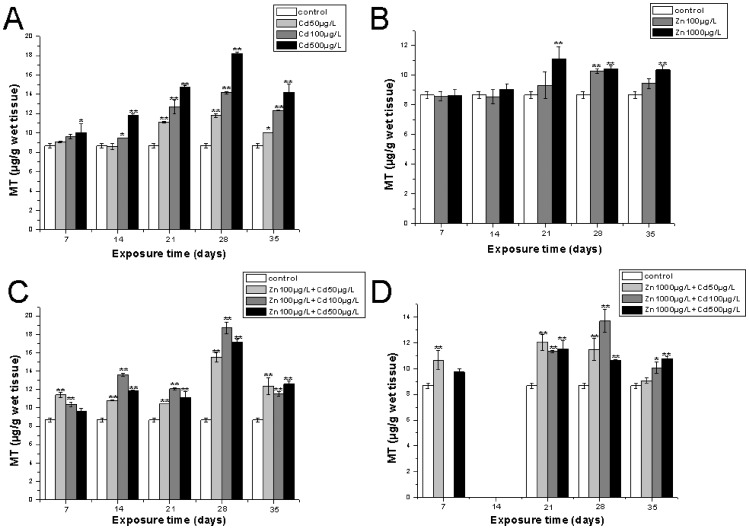
The MT levels in the gills of *S.henanense* exposed to Cd and Zn as either single or both metals combined for 7, 14, 21, 28, or 35days. Data were expressed as mean values ±standard error of four animals. Statistical significance was denoted by *P<0.05, **P<0.01 compared to the respective control crabs.

**Figure 4:**
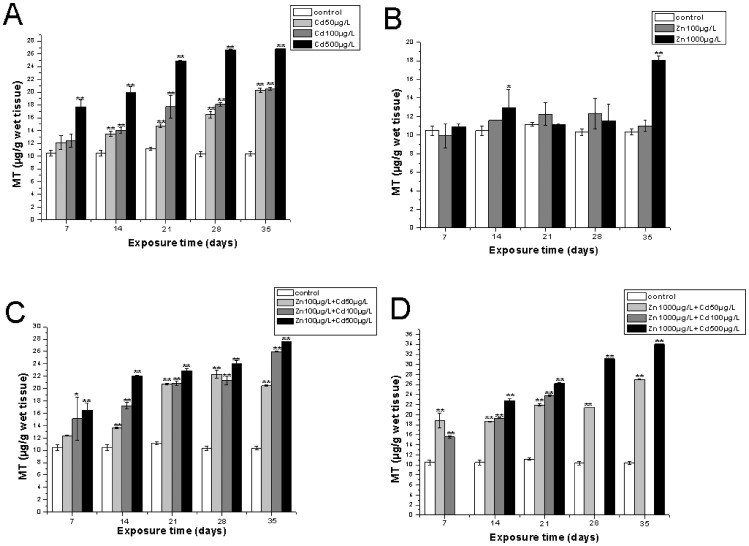
The MT levels in the hepatopancreas of *S.henanense* exposed to Cd and Zn as either single or both metals combined for 7, 14, 21, 28, 35days. Data were expressed as mean values ±standard error. Statistical significances were analyzed using one-way ANOVA compared to the control, *P<0.05, **P<0.01.

The effects of single metal and metal mixture on MT induction in the gills and hepatopancreas are shown in Fig.3 and Fig.4, respectively. The MT induction pattern was metal-specific, and the different patterns of MT induction were shown between the hepatopancreas and gills and were clearly tissue-specific. In the treatment of crabs exposed to Cd only, including 50 µg/L, 100 µg/L, 500 µg/L Cd, there was a time- and dose-dependent increase in the levels of MT induced in the gills until day 28 when the highest value reached 18.22±0.11 µg bound Cd/g wet tissue. However, after exposure for 35 days, the amount of induced MT decreased to 14.21±0.82 µg bound Cd/g wet tissue in the exposure of 500 µg/L Cd (Fig.3A). In the treatment with Cd alone, the amount of MT induced in the hepatopancreas increased sharply during the whole experimental period, and reached the highest value of 26.78±0.044 µg/bound Cd/g wet tissue in the exposure of 500 µg/L for 35 days (Fig.4A). It was very interesting that the dose- and time-dependent increases that we observed in the results of the Cd treatment were not obvious in the results of MT induction within the gills and hepatopancreas of crabs exposed to Zn alone (Fig.3B, Fig.4B). The induced MT reached highest values of 18.71±0.60 µg/bound Cd /g wet tissue in the treatment of 100 µg/L Zn+100 µg/L Cd for 28 days, which was higher than the MT induction by corresponding single Cd pollution. The MT levels decreased when crabs were exposed for 35 days(Fig. 3C), whereas the MT levels reached highest values of 13.70±0.89 µg/bound Cd /g wet tissue for 28 days and also decreased when exposed for 35 days in the treatment of different Cd doses combined with 1000 µg/L Zn in the gills of *S. henanense* (Fig.3D). However, the amount of MT accelerated till the end of the experimental regime within the hepatopancreas and emerged with highest values of 27.60±0.022 and 34.05±0.11 µg/bound Cd /g wet tissue in the treatment of 100 µg/L Zn+500 µg/L Cd and 1000 µg/L Zn+500 µg/L Cd, respectively (Fig.4C, Fig.4D), which showed higher levels of MT induction compared to the treatments with Cd alone. It was interesting that the mode of MT induction in the metal mixture group appeared to be similar to the single Cd treatment group.

Throughout the experimental period, the amounts of MT appearing in the control group were steady at 8.67±0.22 µg/bound Cd /g wet tissue in gills and 10.46±0.48 µg/bound Cd /g wet tissue in the hepatopancreas.

## Discussion

The toxic effects of heavy metals result from the generation of reactive oxygen species causing significant molecular damages within organisms [28]. Cd and Zn are related heavy metals, which are often present simultaneously as environmental contaminations. It has become more and more important to study the mechanisms of toxic action in order to obtain a better understanding of the interaction of the two metals. Cd causes the production of reactive oxygen species (ROS) and disturbs the antioxidant defence system. MDA is a major end product of lipid peroxidation (LPO), and an index of ROS peroxidation. It cross-links proteins, DNA, and nucleotides at the same or opposite strands. In the present study, the toxicity of heavy metals led to an increase of MDA content, resulting from the peroxidation of membrane polyunsaturated fatty acids (PUFAs) [28], [29]. Lipid peroxidation was more obvious in Cd-treated animals than with Zn. Additionally, our results showed that MDA levels were enhanced both in gills and hepatopancreas with the rise of Cd concentration and time exposure. It displayed time- and dose-dependence which is in agreement with our previous studies on acute Cd exposure [7]. In the single Zn treatment group, the MDA content increased also with the rise of exposure concentration and exposure duration. However, the MDA content did not increase significantly compared to the control group at the lower Zn concentration in the hepatopancreas. Similarly, Wu et al. [30] showed that the white shrimps, *Litopenaeus vannamei,* were able to repair the hepatopancreas damage caused by lower Zn treatement from histopathological observations. Nevertheless, the increase of MDA levels were clearly shown to be tissue-specific. The MDA contents in the gills increased significantly from exposure for 14 days to 35 days in the animals with lower Zn-treatment. Overall, these results suggest that the gills may be more sensitive to metal-induced oxidative stress than the hepatopancreas [31]. Generally speaking, in the metal mixture group the MDA concentrations decreased compared to single Cd exposure. Zn itself could be involved in the protection against oxidative stress [32]. Zn may function in the protection from free radical damage due to several reasons. Zn may 1) maintain an adequate level of MTs, which are free-radical scavengers at the same time; 2) be an essential component of Cu,Zn-SOD; 3) be a protective agents for thiols and other chemical groups [32]. It is noteworthy that the MDA contents increased at lower Zn-concentrations and decreased at higher concentrations in the gills. This phenomenon is in good agreement with the work on *Porcellio scaber* by Zidar et al. [33]. When animals were exposed to a mixture of Cd and Zn, lower concentrations stimulated the assimilation of both metals while higher concentrations significantly reduced Cd assimilation. This is possible because in the exposure to a Cd and Zn mixture, Zn interfered with the Cd transport system and lower uptake may be due to a saturation of the transport system or increased excretions, so via feces [33], [34]. In the hepatopancreas the MDA contents showed dose- and time-dependence in the lower metal mixture group (different Cd exposures adding with 100 µg/L Zn) which also indicated clearly tissue-specific effects.

The protective role of Zn from cellular toxicity of Cd was shown by various studies [35], [36]. Our data of MDA contents adds additional evidence to this. That may be because of similar physiochemical characteristics of Cd and Zn which makes Cd to substitute Zn in antioxidant enzymes and antioxidant substrates (e.g. metallothionein-like proteins) [37]. Additionally, Cd and Zn are both potential inducers of MTs [38], [39], [40] and it has been reported that MTs play a very important role in the interaction of Cd and Zn [41]. In addition, as mentioned above MTs have the ability of removing free radicals. For this reason the MT contents were measured in our present study. The results indicated that MTs concentration increased after single Cd exposure and showed a time- and dose-dependence which was clearly tissue-specific. This is in accordance with our previous studies on acute Cd exposures as shown by Ma et al. [8]. However, the MT induction pattern was also clearly tissue-specific after a single Cd exposure. With the extension of exposure time, the MT induction was delayed, especially at higher doses in the gills (Fig.3A). This might be due to an increased toxicity of Cd in freshwater crabs, resulting in a decreased MT content. Long-term exposure and high dose has an impact on the normal structure and function of some organs, leading to a change of normal metabolism and physiology, affecting the ability of oxygen respiration, morphology and function of the intestinal tract, and a reduction of protein synthesis in cells [42], [43], [44]. Otherwise, gills are the main entry sites of metals, and also as a transient metal storage pool, the bioaccumulation of metals in the gills depended on the interactions of absorption, retention and excretion [44], [45], [46]. So the MT content changed accordingly. In the hepatopancreas, the MT induction seemed to be saturated and without a significant change from 28 days to 35 days (Fig.4A), which indicated that hepatopancreas had a higher Cd-bearing ability than gill. Unlike Cd, Zn showed a limited ability to induce the synthesis of denovo MT synthesis, the maximum concentration of induced MT within tissues at higher Zn levels for 35 days was much lower than the maximum level of MT induction by the Cd exposure groups (Fig.3B; Fig. 4B). These results showed different patterns of MT induction by Cd and Zn. As an ubiquitous and essential element for organisms, Zn has the ability to induce the synthesis of MT, which plays a key role in the regulation of Zn metabolism, including absorption and storage. On the other hand, according to Morgan and Morgan [47], physiological pathways should exist for the physiological control of this element in the tissues in a wide range of Zn concentrations till the Zn content or the exposure time exceeds causing a collapse of the regulatory system. Thus the authors considered that the forepart of Zn exposure response in the present study was likely due to its regulatory mechanisms where MT should be included. Whether in the gills or in the hepatopancreas, the results of our quantitative analysis of MDA and MT indicated that their effect combined metal exposure is not a simple addition of single metal exposure effects. However, it appeared that the metal mixture response was dominated by the Cd effect, which is similar to the work on rainbow trout by Lange et al. [40]. This is possible due to the fact that Cd has a higher affinity for thiol groups than Zn [48], [49] and thus Cd could displace Zn from its complex with MT [50]. On the other hand, MT binds with Zn at a faster rate than with Cd. Zn also stays MT-bound in the cytosol for less time than Cd, and then appears in lysosomes degrading MT, and dose not induce an additional synthesis of MT [46], [51], [52], [53].

It remains controversial whether MT is appropriate as a biomarker for environmental heavy metal pollution. According to the suggestions given by Pederson et al. [17], three important issues need to be considered for MT being used as a biomarker: 1) whether the biomarker concentration in tissues could reflect chronic levels of environmental pollution; 2) whether the biomarker contents in specific tissues could reflect the pollution extent of these tissues; 3) whether different biomarkers are consistently expressed? Since Zn showed different MT induced behavior with Cd, MT could be considered cautiously as a biomarker.

Above all, the results in our study indicated that the contents of MDA and the concentrations of MTs were complementary to each other. The authors thus suggest to use a suite of biomarkers in the monitoring of heavy metal pollution in the aquatic environment. Due to similar physiochemical properties of Cd and Zn, these metals can interact with each other through the substitution for the enzyme binding Zn and competition for protein binding sites such as metallothionein-like proteins. Possible results are, therefore, reflecting the interactions of various factors.

## Conclusions

Examing only one kind of biomarker may not always be sufficient in order to monitor the full extent of heavy metal pollution. When investigating the changes of MDA levels and MT contents after single and co-exposure of Cd and Zn, our results showed that MDA contents decreased in the metal mixture compared with single metal exposures and MT plays a key role in the process of Zn protection of Cd toxicity. Different induction patterns are shown in the single Cd, Zn and metal mixture groups. In the past have several biomarkers only separately been applied in the monitoring of heavy metal pollution. The authors suggest here that it would be appropriate to use them in a combined way, or even as a suite to detect the extent of heavy metal pollution in the aquatic environment.
